# Experimental and Computational Investigation of Surface‐Responsive Riboflavin‐Based Self‐Assembled Systems

**DOI:** 10.1002/chem.202500726

**Published:** 2025-09-15

**Authors:** Ruth Aizen, Thangavel Vijayakanth, Sarah Guerin, Pierre‐André Cazade, Om Shanker Tiwari, Bin Xue, Linda J. W. Shimon, Yi Cao, Damien Thompson, Ehud Gazit

**Affiliations:** ^1^ The Shmunis School of Biomedicine and Cancer Research George S. Wise Faculty of Life Sciences Tel Aviv University Tel Aviv 6997801 Israel; ^2^ Department of Materials Science and Engineering Tel Aviv University Tel Aviv 6997801 Israel; ^3^ Department of Physics Bernal Institute University of Limerick Limerick V94 T9PX Ireland; ^4^ National Laboratory of Solid‐State Microstructure Department of Physics Nanjing University Nanjing 210000 China; ^5^ Department of Chemical Research Support Weizmann Institute of Science Rehovot 76100 Israel

**Keywords:** H‐bonding, mechanical properties, metabolites, Riboflavin, Self‐assembly

## Abstract

Metabolites, including amino acids, nucleobases, and vitamins, have emerged as promising candidates for sustainable functional materials due to their inherent biocompatibility and low fabrication costs. Notable examples include glycine‐based nanogenerators, indigo‐based organic transistors, and caffeine‐based optical waveguides. Riboflavin (vitamin B2), forms optically active supramolecular structures in the *tapetum lucidum* of lemurs and cats; however, its detailed packing and functional role remain unknown. Here, aiming to explore the bio‐inspired self‐assembly of riboflavin to uncover potential device applications, we discovered and extensively characterized a new single co‐crystal using a combination of crystallography, microscopy, and mechanical experiments supported by atomistic molecular models to understand the organization on different surfaces. The crystals exhibit pronounced surface responsiveness, leading to the formation of distinct branched, twisted, and serrated micron‐scale morphologies as the riboflavin self‐assembled on different substrates of copper, mica, and silicon. This intrinsic ability to adapt shape and generate substrate‐templated structures was confirmed computationally and experimentally and was attributed mainly to the crystal's relatively low Young's modulus, reflecting its lattice flexibility. This structure–function study of an adaptable metabolite crystal offers fundamental insights into how molecular organization governs mechanical responsiveness, advancing the understanding of bio‐inspired crystallization and paving the way for future technological applications.

## Graphical abstract

A self‐assembled crystal of riboflavin exhibits responsive modes of surface‐directed molecular assembly, accompanied by measurable electromechanical properties. This behavior highlights how subtle variations in molecular packing and supramolecular interactions at the crystal interface can directly influence macroscopic functional responses. By bridging the molecular organization with emergent physical properties, these findings elucidate key structure–function relationships, offering new perspectives for the design of bioinspired materials with tunable electromechanical properties.

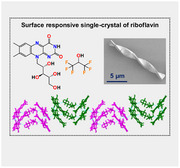

## 1 Introduction

Self‐assembly in biological systems enables the spontaneous organization of individual molecules into a diverse range of supramolecular structures and architectures. Nature exploits this process to assemble simple building blocks into intricate biological materials and processes that drive life.^[^ ^1^ ^]^ Governed primarily by noncovalent binding, these supramolecular assemblies exhibit dynamic properties due to the reversibility of the individual interactions, which makes the materials responsive to external stimuli such as light, electric field, pH and more.^[^ ^2^ ^]^ In nature, self‐assembling materials originate from diverse molecular building blocks, including proteins, nucleic acids, lipids, and metabolites such as amino acids, nucleobases, and vitamins.^[^ ^3^ ^]^

The versatile and readily tunable physical properties of metabolites and their derivatives, as well as their inherent biocompatibility, biodegradability, and low cost, make them promising candidates for diverse applications as sustainable materials in current and emerging technology applications, from the fabrication of optical materials to the development of high‐performance electronics.^[^ ^3b,c^ ^]^ For instance, glycine, a single amino acid, exhibits remarkable, relevant to technology piezoelectric properties in certain polymorphs of glycine crystals, where efficient molecular packing along specific crystallographic planes and directions results in piezoelectricity values as large as 200 pC/N.^[^ ^4a^ ^]^ Coupled with the inherently low dielectric constant of < 5 for organic assemblies, the electromechanical responses produces significant voltage outputs. For example, up to 0.45 V for γ‐glycine amino acid^[^ ^4a^ ^]^ and up to 1.4 V for acetylated tryptophan amino acids,^[^ ^4b^ ^]^ potentially bridging the gap between biological and inorganic materials for piezoelectric devices. Similarly, indigo, a dye derived from plant leaves, exhibits intriguing characteristics owing to its low solubility and high melting point, which arise from inter‐ and intra‐molecular hydrogen bonding (H‐bonding). The efficient charge transport observed in highly ordered thin films of indigo suggests its potential for the fabrication of organic field effect transistors.^[^ ^5^ ^]^ A final example is the construction of passive optical waveguiding from tubular crystals of caffeine, a natural noncanonical nucleobase, highlighting the feasibility of utilizing natural and biodegradable compounds in electro‐optical applications.^[^ ^6^ ^]^ The discovery of the ability of simple natural building blocks to assemble into complex functional architectures underscores the importance of understanding and manipulating crystal structures at the nanoscale level. Such endeavours hold immense potential for advancing next‐generation optoelectronic devices, including solar cells, light‐emitting diodes, and power generation devices.

Here, we studied riboflavin, primarily recognized for its role as an essential water soluble vitamin involved in various metabolic pathways.^[^ ^3c^ ^]^ Interestingly, it has been discovered to form optically functional supramolecular structures within the *tapetum lucidum* of lemurs and cats. The *tapetum* is a reflecting layer found behind the retina and is responsible for reflecting back unabsorbed light.^[^ ^7^ ^]^ These structures, consisting of plate‐like crystals in lemurs and fluorescent rodlet‐like structures in cats, enhance vision in low‐light conditions by facilitating secondary light absorption.^[^ ^8^ ^]^ However, the precise biogenic crystal structures remain unknown. Although riboflavin is widely studied^[^ ^9^ ^]^ and there are a few known synthetic polymorphs of riboflavin crystals (un‐solvated),^[^ ^9a^ ^–^ ^d^ ^]^ its crystal structure was only recently reported, as determined from synchrotron powder X‐ray Diffraction (XRD) data in conjunction with periodic DFT‐D calculations for geometry optimization.^[^ ^9e^ ^]^ In contemporaneous work, a multitechnique approach, including microcrystal XRD, powder XRD, 3D electron diffraction (3D‐ED), high‐resolution solid‐state ^13^C‐NMR spectroscopy and dispersion‐augmented density functional theory (DFT‐D) calculations, was employed to study the solid‐state structure of the same polymorph of riboflavin.^[^ ^9f^ ^]^ However, a different intermolecular H‐bonding arrangement was found,^[^ ^9f^ ^]^ as compared to the published structure of riboflavin from Powder X‐ray Diffraction (PXRD) data.^[^ ^9e^ ^]^ Here, our study on the crystallization of riboflavin was motivated by the lack of structural information on its biogenic crystal form and possibility that different natural crystal morphologies may correspond to distinct crystal structures. Acknowledging the known challenges in obtaining single crystals of riboflavin, we carried out crystallization experiments using the solvent hexafluoro‐isopropanol (HFIP). This approach enabled us to obtain a new Single‐Crystal X‐ray Diffraction (SCXRD) structure of riboflavin. While previous studies have reported the structure of riboflavin in its un‐solvated form,^[^ ^9e–d^ ^]^ our work presents the crystal structure of a riboflavin/HFIP solvate. Importantly, we find a profound difference in the flexibility and macroscopic structure of the crystals as templated by the crystal–substrate interface and solvent environment. These findings advance efforts to harness the remarkable properties of self‐assembling metabolites for diverse technological advancements.

## 2 Results and Discussion

### 2.1 Self‐assembly and Structural Characterisation of Riboflavin/HFIP Crystals

**Figure 1:**
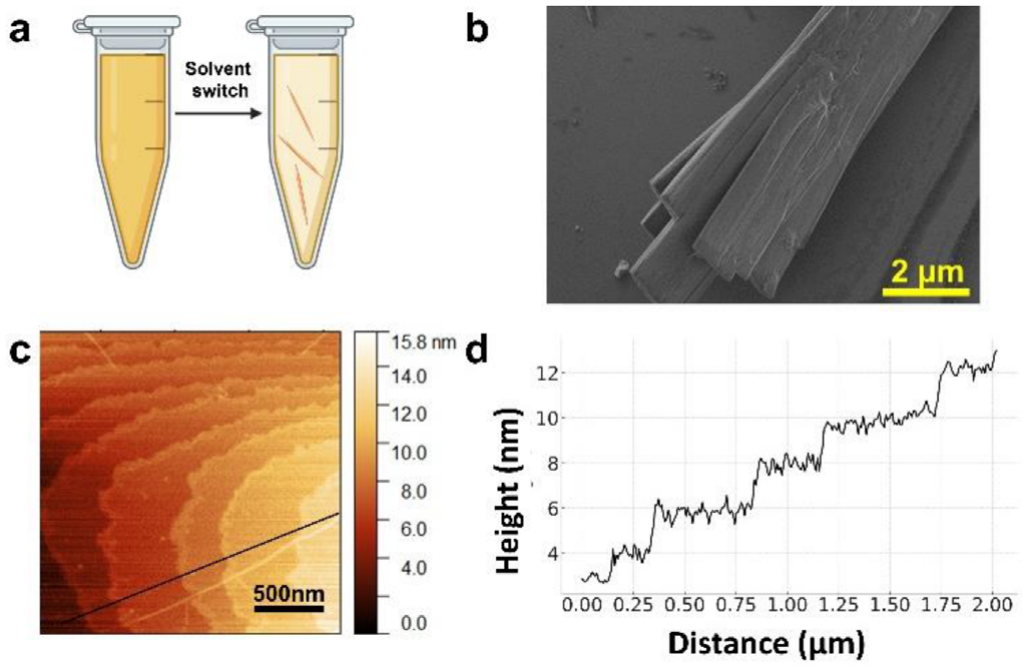
a) Schematic illustration of the riboflavin/HFIP self‐assembly process using the solvent switch method. b) HR‐SEM image of the crystals. c) AFM surface analysis of the crystal on a mica substrate. d) Cross‐section analysis displaying the height profile drawn in image (c).

Our initial goal was to crystallize riboflavin directly in water. However, the limited solubility of riboflavin in water results in rapid aggregation, forming a powder unsuitable for SCXRD analysis. To address this issue, we employed the solvent switch method. Riboflavin was dissolved in 1,1,1,3,3,3‐hexafluoroisopropanol (HFIP) at a high concentration followed by dilution in water (Figure 1a). HFIP is a versatile solvent often used to dissolve peptides and polymers that are insoluble in common organic solvents.^[^ ^10^ ^]^ In a 4% HFIP solution, needle‐like orange crystals became apparent after several days. Figure 1b displays the high‐resolution scanning electron microscopy (HR‐SEM) image of the crystal and Figure 1c illustrates the atomic force microscopy (AFM) topography analysis, revealing a laminated structure with each layer measuring approximately 1 nm thick.

To gain deeper insights into the higher‐order crystal packing and the modes of H‐bonded interactions at the atomic level, single crystals of riboflavin were comprehensively studied using SCXRD analysis. Detailed single crystal data collection and refinement parameters, bond distances, bond angles and torsion angles of the riboflavin crystal are summarized in Table S1‐S5.

**Figure 2:**
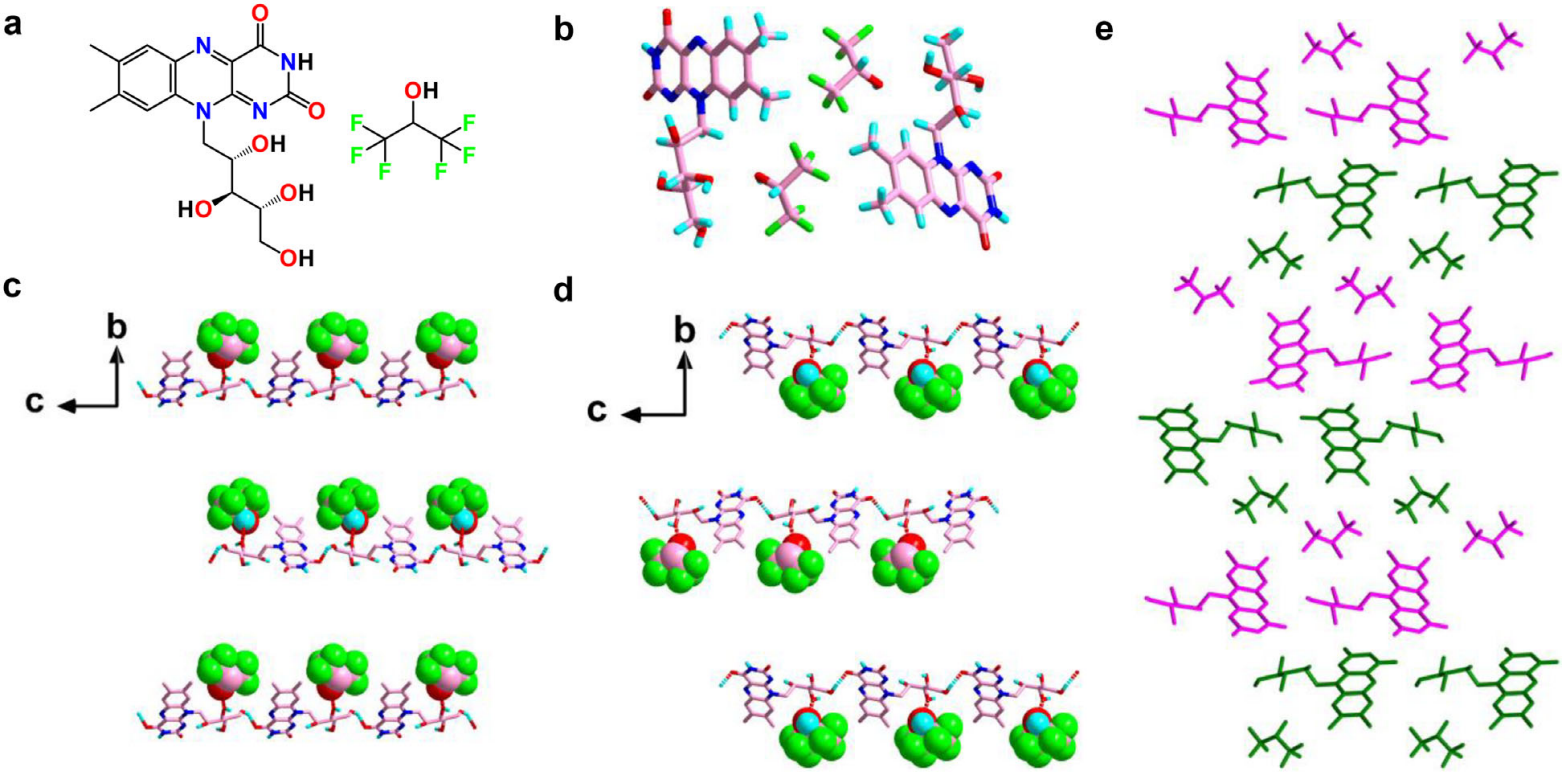
a) Chemical structure and b) asymmetric unit of riboflavin and HFIP. (c and d) View of the H‐bonded 1D network structure of c) RF1 and d) RF2 along the crystallographic a‐axis. The highlighted space‐filling model illustrates the molecular arrangement of the co‐crystallized, ordered HFIP solvent molecules. The carbon, hydrogen, nitrogen, oxygen, and fluorine atoms are colored in rose, turquoise, blue, red, and green, respectively. e) Representation of the cis‐oriented structural arrangement of RF1 and RF2 along the crystallographic a‐axis that creates the “herringbone” stacking mode. The cis‐oriented conformational arrangement of RF1 and RF2 is highlighted in green and pink color.

The Oak Ridge Thermal Ellipsoid Plot (ORTEP) analysis illustrated the thermal behavior of the riboflavin molecules through ellipsoid representations, showcasing their similar sizes, shapes, and consistent orientations (Figure S1). The crystal exhibited a monoclinic unit cell symmetry with the noncentrosymmetric space group *P*2_1_ and the following lattice parameters: a = 5.43380(10) Å, b = 35.5503(8) Å, c = 11.9651(3) Å, α = γ = 90°, β = 101.177(2)° and V = 2267.5 Å^3^. The molecular structure of riboflavin and co‐crystallized HFIP is shown in Figure 2a. The asymmetric unit contains two different riboflavin molecules (designated RF1 and RF2) and two equivalent HFIP solvent molecules (Figures 2b and S2). The packing diagram displays two equivalents of RF1 and RF2, alongside four equivalents of HFIP molecules (Figure S3). Both RF1 and RF2 formed extensive and multiple directional supramolecular H‐bonded assemblies. Each riboflavin molecule was associated with a specific HFIP molecule, with oxygen—oxygen distances of 2.6076(74) Å (RF1, O4···O13) and 2.5770(68) Å (RF2, O10···O14), respectively, as shown in Figure 2c,d. Notably, none of the HFIP acceptor fluorine atoms participated in any significant H‐bonded assemblies with RF1 or RF2. The isoalloxazine ring of each riboflavin molecule interacted with each other, forming an infinite alternate stacking mode of H‐bonded assemblies (Figure S4). Particularly, the RF2 riboflavin isoalloxazine ring was linked with the RF1 riboflavin molecule through three significant intermolecular H‐bond interactions: one between the amino N7 of RF2 and oxygen O2 of RF1, another between the amino N7 of RF2 and oxygen O5 of RF1, and the third between O7 of RF2 and N3 of RF1 (Figure S5). Moreover, the isoalloxazine ring of RF2 also engaged in two intramolecular H‐bond contacts of oxygen—oxygen (O8···O12) and nitrogen—oxygen (N6···O9) (Figure S5). These uncommon H‐bonding interactions of riboflavin are attributed to the high donor and acceptor capabilities of the pyrimidine ring of the isoalloxazine group.

The riboflavin molecule RF1 formed 1D intermolecular H‐bond interaction with the adjacent RF1 molecule via extended chain networks (along the c‐axis) through head‐to‐tail interactions, with a donor–acceptor distance of 2.7694(1) Å (O2···O6), as depicted in Figure 2c. Similarly, the second riboflavin molecule RF2 engaged in H‐bonded contact with the neighboring RF2, resulting in a relatively short 1D molecular arrangement (along the a‐axis), with a donor–acceptor distance of 2.7468(0) Å (O12···O8), (Figure 2d and S5). The evident stacking modes between RF1 and RF2 were significant contributors to maintaining the solid‐state conformational stability. Remarkably, the higher order crystal packing of the RF molecule displayed a cis‐oriented arrangement of head‐to‐tail molecular conformation (see RF1 and RF2 highlighted in green and magenta, Figure 2e), which propagated to assemble in a typical “herringbone” stacking mode. Additionally, the isoalloxazine rings of both RF1 and RF2 were stabilized in the solid‐state packing through parallel aromatic interactions along the ab plane, with a typical C–C distance of 5.3348(1)Å, (Figure S6).

Here, its important to note that the structure of riboflavin reported in our work differs from previously reported structures^[^ ^9e,9f^ ^]^ In the present study, riboflavin crystallized in the monoclinic (*P*2_1_) crystal system, whereas known structures crystallize in the orthorhombic (*P*2_1_2_1_2_1_) system. A key distinction is the presence of two distinct riboflavin molecules in the asymmetric unit, along with two equivalent HFIP molecules. Additionally, the H‐bonding pattern of the terminal OH groups on the side chains (O6 and O12 in our case) reveals strong intermolecular O − H···O hydrogen bonds with neighboring OH groups (O4 and O10) and C = O groups (O2 and O8) from the aromatic ring system. Furthermore, the presence of HFIP molecules plays a crucial role in stabilizing the hydrogen‐bonding networks, a feature absent in previously reported riboflavin structures.

### 2.2 Conformational Change of the Riboflavin/HFIP Crystal on Different Surfaces

**Figure 3:**
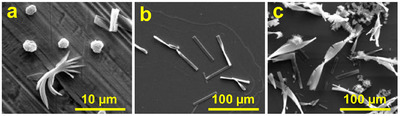
SEM images of riboflavin/HFIP crystal deposited on a) copper, b) mica, c) silicon.

We initially set out to image the crystals using scanning electron microscopy (SEM) and observed that, on silicon wafers, the crystals displayed a twisted morphology. Noting that the silicon surface is relatively hydrophobic, we hypothesized that surface properties might influence crystal morphology. To test this hypothesis, we deposited the crystals on a range of different substrates and found that their micron‐scale structures varied markedly depending on the surface. Specifically, on copper, the crystals split at the edges and developed branched structures; on mica, they exhibited a twisted form; and on silicon, they appeared both twisted and serrated. These distinct morphologies suggested a strong substrate‐dependent growth behavior. To gain further insight into the origin of these variations, we employed additional characterization techniques to investigate the interplay between surface properties and crystal formation (Figure 3). (more SEM images taken on these surfaces can be found in Figure S7)

**Figure 4:**
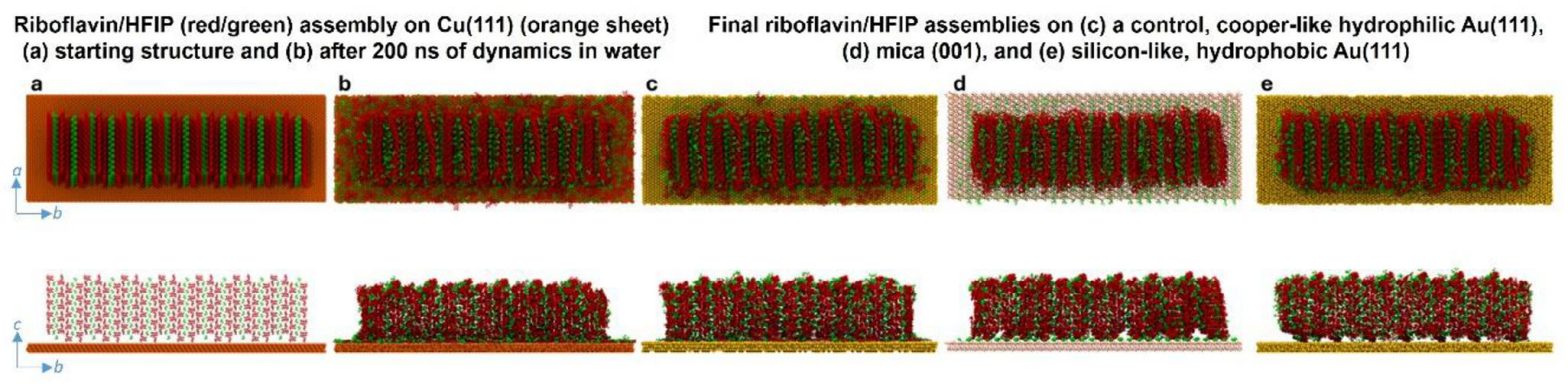
MD simulations of the riboflavin/HFIP crystal on four substrates in water. Riboflavin crystal on copper (111) a) before and b) after 200 ns of molecular dynamics in water. The final structures obtained on c) a further control, cooper‐like hydrophilic gold (111), included for the sake of completion and test of model robustness for capturing hydrophobic versus hydrophilic driving forces. d) mica (001), and e) silicon‐like, hydrophobic gold (111). Upper and lower panels are viewed in the ab‐plane and bc‐plane, respectively. RF and HFIP molecules are colored red and green, respectively.

This intriguing behavior prompted us to employ atomistic molecular dynamics (MD) simulations to further investigate the crystal behavior on various surfaces. Figure 4a‐e shows the starting and final structures of the classical MD simulations in water of the riboflavin/HFIP crystal on Cu(111), on mica (001), and on a Si(111) mimicking surface, namely, the well‐parameterized model hydrophobic bare Au(111) surface. Hydrophobic bare Au(111) surface was chosen to mimic Si(111) as Au(111) has been extensively parameterized and benchmarked for hydrophobic/hydrophilic substrate effects in biomolecule adsorption studies at the water‐surface interface.^[^ ^11^ ^]^ The views shown in Figure 4a‐e are projected in plane and side‐on views along the crystallographic ab and bc planes. For the sake of completeness, we also modeled assemblies on the corresponding model hydrophilic bare Au(111) surface, as an analogue of Cu(111). It is important to note that the nanoscale 11 × 7 × 5 supercell of the crystal unit cell used in our simulations is too small to exhibit the full micron‐scale restructuring observed experimentally. Due to computational limitations, full density functional theory (DFT) calculations on the supercell models are prohibitive, and modeling thermal effects is challenging. Therefore, we rely on classical MD simulations to model larger area, sub‐micron morphological changes. The MD simulations provide insights into the nanoscale dynamics at the interface with the substrate, which, in conjunction with DFT models of the single crystal (described further below), elucidate the underlying formation mechanisms for the various structures observed experimentally. Figure 4a and b shows the results for copper, which is a naturally hydrophilic substrate. Numerous riboflavin and HFIP molecules shifted downwards to adsorb on the copper surface, showing the competitive nature of the molecule–molecule and molecule–surface interactions. These surface‐bound molecules could serve as anchoring points for crystal growth, potentially stabilizing and facilitating the emergence of the experimentally observed split and branched structure at the micron scale. This behavior was confirmed by the analogous hydrophilic model of a gold surface (Figure 4c). In these metal models, the surface is uniformly hydrophilic and does not offer any specific anchoring point for strong pairwise interactions between atomic sites on the riboflavin/HFIP assembly and the metal substrate. The observed branching and splitting of the crystal on copper may help release the strain built around the b‐axis as the crystal grows and the resulting splayed morphologies may redirect the crystal growth away from the alternative twisted morphologies observed on mica and silicon (see SEM images in Figure 3 and models in Figure 4d and e). Figure 4d shows the results obtained for mica as a substrate. Mica is another hydrophilic surface, but unlike copper, it contains charged ions, providing specific anchoring points. On mica, HFIP molecules in contact with the surface at the bottom of the crystal created attractive electrostatic interactions with the ions embedded in the mica surface. This site‐specific anchoring of the crystal on mica induced an internal reorganization in the riboflavin/HFIP assembly as the riboflavin molecules nudged down to stabilize the mica–HFIP–riboflavin interaction. These interactions and structural changes stabilized the crystal and compensated for the shear tension in the crystal (as predicted using the DFT models, below), which is consistent with simple twisting without any branching in the micron scale structure, as experimentally observed. Figure 4e shows the crystal in contact with hydrophobic gold as a model for bare silicon, such as a silicon wafer with no oxide layer.^[^ ^12^ ^]^ On this substrate, there was no downward diffusion of molecules from the crystal, with only very weak, nonspecific van der Waals forces maintaining a loose association between the crystal and the surface. The lack of stabilization from the surface, which may represent a hydrophobic bare silicon substrate, along with the tendency of riboflavin to present complementary core hydrophobic sites to the surface, could enhance the twisting and tearing behavior observed in the micron scale SEM images of riboflavin on silicon and is consistent with the promotion of helicity in the large‐area crystal (discussed more below, see Figure 6).

To further probe the twisting and tearing behavior on hydrophobic surfaces and test the predictions from modelling, we deposited the crystals on Teflon and Siliconised glass (Si‐glass). The results clearly demonstrated that this behavior was enhanced when the surface was hydrophobic, as shown in Figure S8a and b, respectively. On the contrary, when the crystals were deposited on a hydrophilic glass coverslip, almost no twisting was apparent, as depicted in Figure S8c.

To obtain quantitative insight into the molecular driving forces behind the observed substrate‐induced reconfigurations of the molecular self‐assemblies, the interaction energy was calculated between the crystal components (riboflavin and HFIP) and the substrate, together with the internal riboflavin–HFIP interactions in the crystal (Figure S9). The hydrophobic gold surface, as a model for bare silicon, did not significantly interact with either riboflavin or HFIP molecules. As a result, the internal propensity of the crystal for twisting (as predicted using DFT, below) was not compensated by the surface, potentially leading to the extreme twisting and tearing observed in the SEM images. Conversely, copper interacted strongly with both riboflavin and HFIP, weakening the interactions between these molecules. The strong binding to copper also weakened the riboflavin self‐interactions, namely the van der Waals and electrostatic interactions between riboflavin molecules in the crystal. The HFIP self‐interaction was strengthened to balance the forces in the crystal–surface complex, as the copper became coated in a thin, randomly distributed layer of HFIP molecules (Figure 4a,b). Similarly, mica interacted with HFIP molecules to form an ordered and regularly spaced layer of surface‐bound HFIP molecules. However, unlike copper, mica did not interact strongly with riboflavin. The riboflavin molecules in the bottom layer closest to the mica surface simply nudged down slightly in response to the HFIP diffusion onto the surface, as shown in Figure 4d. The HFIP hydroxyl group showed a strong affinity for the charged mica surface and the resulting loss of HFIP molecules from the crystal led to a decrease in HFIP self‐interactions and weakened its interactions with riboflavin. Hence, the riboflavin molecules rearranged in the crystal to compensate for the gap left by the now surface‐bound HFIP molecules. Taken together, the simulations and experiments demonstrate rational modulation of crystal assembly microscale morphology by balancing the internal crystal binding forces and crystal–substrate interaction strengths.

### 2.3 Mechanical Properties and DFT Analysis of Riboflavin/HFIP

The observation of the crystal's ability to bend and twist, coupled with its noncentrosymmetric monoclinic symmetry, inspired our interest in investigating its electromechanical properties. Noncentrosymmetric compounds are particularly intriguing due to their symmetry‐dependent properties, such as piezoelectricity, ferroelectricity, and second‐order nonlinear optical behavior, which enable numerous nanotechnology applications.^[^ ^13^ ^]^ Piezoelectric materials generate electrical energy in response to mechanical deformation and vice versa.^[^ ^14^ ^]^ Recently, piezoelectricity has been widely reported in several natural materials including bone, collagen, viruses, cellulose, peptides, and single amino acid assemblies.^[^ ^4^, ^13^, ^15^ ^]^ To investigate the electromechanical properties of riboflavin/HFIP crystals, we employed a combination of experimental and theoretical studies.

**Figure 5:**
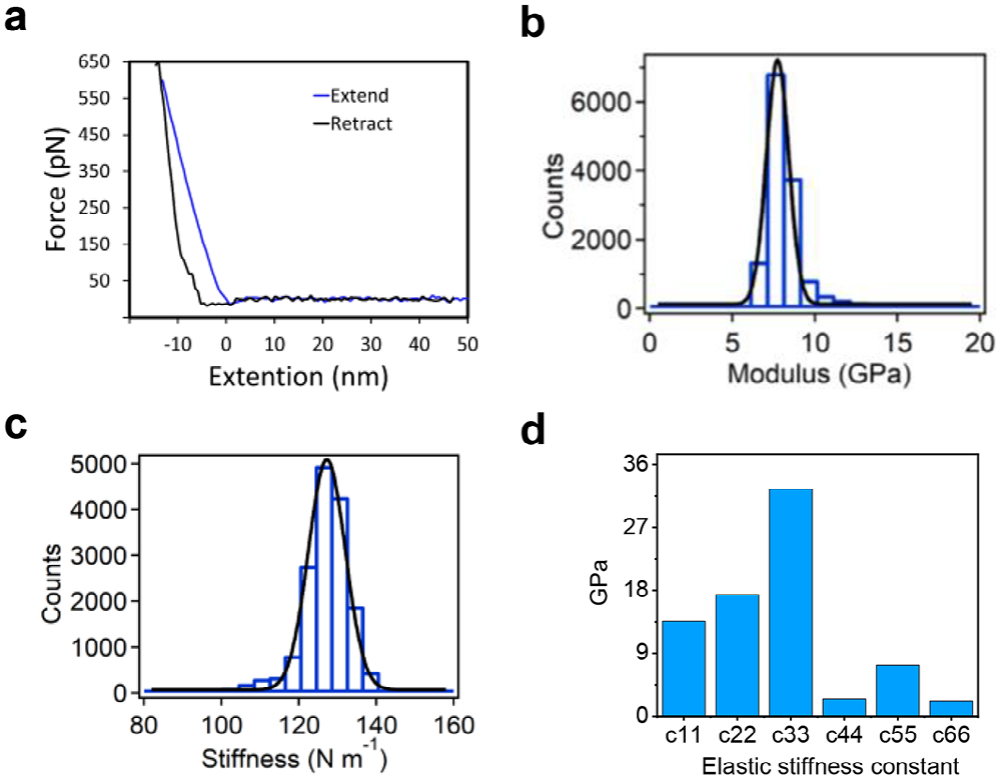
a) Typical force–distance traces. The blue line corresponds to the fitting of the contact region in the “extend” trace using the Hertz model. b) Statistical distribution of the obtained Young's modulus. c) Statistical distribution of point stiffness measurements. The black line in b and c corresponds to the fitting using a Gaussian model. d) Elastic constants of riboflavin/HFIP crystals predicted by DFT calculations.

First, we examined the micromechanical properties of the crystals to understand whether there is an intrinsic ability of the crystal to bend and twist. For this purpose, we conducted AFM nanoindentation measurements. AFM nanoindentation is a technique that uses an atomic force microscope to measure mechanical properties at the nanoscale, such as elastic modulus, hardness, adhesion, and viscoelasticity. In this method, a sharp tip attached to a flexible cantilever is brought into contact with the sample surface and pressed into it with controlled force. A typical force–distance trace in AFM nanoindentation represents the measured interaction force as a function of the tip–sample separation during approach and retraction (Figure 5a). By analyzing this curve with contact mechanics models (e.g., using Hertz model – see Materials and Methods Section in the Supporting Information), key properties such as Young's modulus and indentation depth can be extracted. AFM nanoindentation allows high‐resolution, localized measurements on small or soft materials such as polymers, thin films, or biological tissues and can be performed in various environments, including air, liquid, or vacuum. It is particularly useful for characterizing nanostructures and heterogeneous materials while simultaneously imaging surface topography.^[^ ^16^ ^]^ The results are depicted in Figure 5a‐c. In these experiments, crystals were carefully fixed to a flat glass coverslip, as illustrated in Figure S10. The cantilever was set to approach and retract from the surface of the crystals at a constant speed of 2 µm s^−1^.

The Young's modulus (the modulus of elasticity) of the crystal at a given position could be calculated by fitting the approaching traces using the Hertz model (see Materials and Methods in the Supporting Information). Moreover, the point stiffness could then be calculated according to the force–distance traces. The Young's modulus of the crystals was found to be 7.7 ± 0.5 GPa (Figure 5b), with a statistical point stiffness of 127.6 ± 3.3 N m^−1^ (Figure 5b, c). These results indicate that the Young's modulus is low, confirming weak intermolecular forces between molecules. Thereby allowing for relatively easy deformation of the crystal as a whole, as demonstrated when the crystal is deposited on different surfaces, inducing shear stress on the crystal. However, within the crystal structure, certain regions or points may exhibit high stiffness due to other specific intermolecular interactions or molecular arrangements that make those points more resistant to deformation.

Following the micromechanical analysis, we sought to investigate the potential structure‐function relationship by employing DFT calculations. The monoclinic symmetry of the riboflavin/HFIP crystals allows for eight nonzero piezoelectric tensor components. The riboflavin/HFIP crystal showed generally low charge tensor components, with one moderate value of e_14_ = ‐0.07 C/m^2^ (Figure S11). The crystal demonstrated a relatively low average Young's modulus value of ∼7.6 ± 3.8 GPa (Table S6), consistent with its experimentally observed twistability, and in excellent agreement with the measured values from AFM nanoindentation presented in Figure 5b. Moreover, the crystal exhibited a significant c_33_ elastic stiffness constant of 33 GPa (Figure 5d). The crystal needs one mechanically strong axis to facilitate twisting, which is the 3 axis for riboflavin. The low shear stiffness resulted in a high predicted maximum piezoelectric strain constant d_14_ = 28 pC/N. The predicted relative permittivity was 4.1 (Table S7), resulting in a maximum piezoelectric voltage constant of g_14_ = 0.55 V m/N. The predicted longitudinal piezoresponse was low with d_22_ = 0.5 pC/N, suggesting that current and voltage outputs may be low in energy harvesting experiments compared to other biomolecular crystals.^[^ ^4b^ ^]^ The overall low values of the charge tensor align with the nonzwitterionic nature of riboflavin, resulting in reduced net charge generated under applied force.

The low shear stiffness arises from a monoclinic angle of more than 100° in the crystal, the presence of small flexible solvent molecules, and the flexibility of the hydroxylated sidechain. The axis of the highest mechanical stability aligns parallel to both the riboflavin and HFIP molecular backbones and the plane of the cyclic motifs. Macroscopically, with the b‐axis being the growth axis of the crystal, the weak shear elastic tensor components along a and c (c_44_ ≈ c_66_ ≈ 2.4 GPa, Figure 5d) make it possible for the crystal to twist as observed in the micron scale SEM experiments and in the nanoscale MD simulations. The weak shear elastic tensor components along a and c can be explained structurally when examining the crystal structure motifs along these axes. The crystal exhibits a herringbone stacking mode, a common motif in organic crystallography.^[^ ^17^ ^]^ The herringbone pattern entails a staggered arrangement of molecules forming a zigzag pattern (Figures 2e and S6). The propensity of the crystals to twist and bend can be attributed to their unique structural characteristics. The herringbone arrangement introduces a degree of flexibility into the crystal lattice (as demonstrated by DFT), allowing it to deform under stress, such as deposition on a surface. Moreover, the directional intermolecular forces that bind the crystal together result in an uneven distribution of stress when deposited on a surface. Here, the H‐bonds along the c‐axis between riboflavin and HFIP (Figure 2c,d), as well as the π‐stacking modes of the isoalloxazine rings of riboflavin, contribute to the degree of flexibility in the herringbone structure. This uneven stress distribution can induce the crystal to bend or twist in a particular manner to release tension.

**Figure 6:**
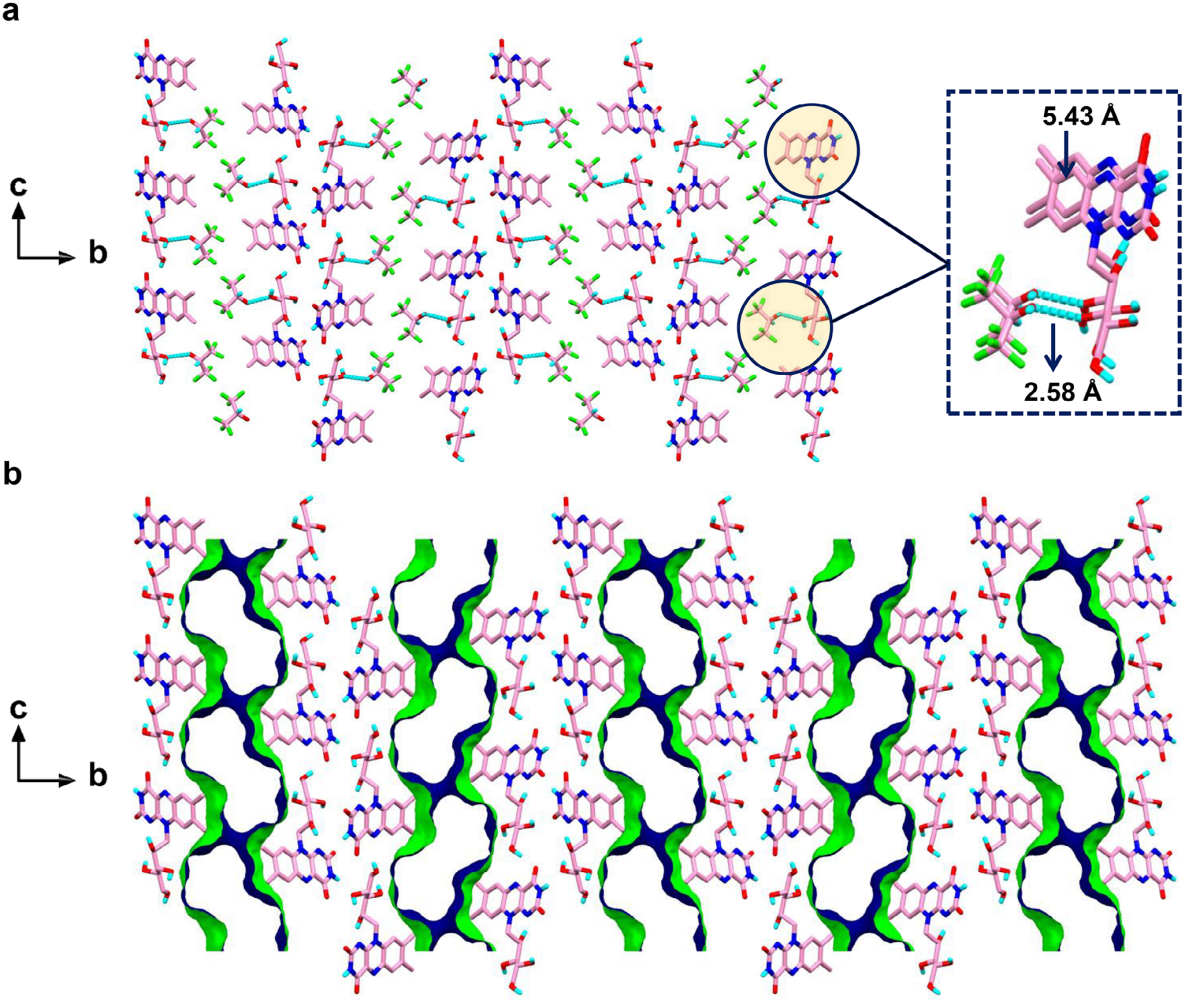
a) Highlighted rare H‐bonds between Riboflavin and HFIP and extensive π‐stacking between the isoalloxazine rings of riboflavin. b) Hydrophobic core created by fluorine atoms in HFIP molecules and the methyl groups of isoalloxazine rings. HFIP molecules are omitted for clarity.

The final identified design element is the presence of mobile HFIP molecules in the structure, situated between the layers of riboflavin, which contributes to the overall degrees of freedom. These HFIP molecules exhibit minimal interaction with each other and form only weak H‐bonds with riboflavin (Figure 6a). The HFIP layer forms a core hydrophobic site for riboflavin to coordinate its hydrophobic methyl groups of the isoalloxazine ring (Figure 6b). It was previously shown that clusters of HFIP molecules associated with the hydrophobic surface of an α‐helix in proteins mimic the interior of a folded protein further inducing the helix formation.^[^ ^10^ ^]^ Consequently, when the crystal twists on a hydrophobic surface, it collapses in a manner that conceals the hydrophobic portion similar to the formation of α‐helix in proteins.

## 3 Conclusion

In conclusion, we demonstrated that the simple metabolite, riboflavin can self‐assemble to form surface‐responsive crystals. Both the nature and structure of the substrate play a role in the behavior of the riboflavin crystal. DFT calculations reveal an inherent twisting propensity prior to substrate contact, driven by the low shear components of the elastic tensor. This is reflected in the herringbone structure and weak supramolecular interactions along the crystal a and c axes. MD simulations on uniform hydrophobic surfaces (e.g., hydrophobic gold, or bare silicon) indicate unstable binding, resulting in extreme twisting as observed by SEM due to the crystal's uncompensated low shear elastic constant, as the crystals split at the edges and formed branches. Conversely, uniform pure‐metal hydrophilic surfaces (e.g., copper) attract and partially unravel crystals without specific pairwise interactions. Physisorbed molecules act as anchor points, releasing tension built by the shear elastic force acting on the crystal as it is twisted and serrated. In a third distinct interaction mode, heterogeneous hydrophilic surfaces such as mica provide specific anchoring points and favorable electrostatic interactions, shaping a mildly twisted crystal morphology. Predictive modelling coupled with systematic testing of the piezoelectric response as a function of film thickness would be an interesting avenue for follow‐up work on this and other metabolite/biomolecule‐based assemblies.^[^ ^18^ ^]^ Understanding electromechanical properties and structure–function relationships of the riboflavin crystal advances metabolite‐based material design. Here, incorporation of HFIP solvent into the crystal structure is crucial in achieving this surface responsiveness and elasticity. The noncentrosymmetric nature of the riboflavin/HFIP crystal, suggests that future exploration of the crystal second harmonic generation properties could further elucidate its electromechanical properties and response to diverse surfaces, which also makes these crystals promising candidates for integration into flexible nonlinear optical devices, including miniaturized frequency converters and tunable photonic circuits.

## Conflict of Interest

The authors declare no conflict of interest.

## Supporting information



Supporting Information

Supporting Information

## Data Availability

The data that support the findings of this study are available from the corresponding author upon reasonable request.
