# Supplementary material for: Experimental and Computational Investigation of Surface‐Responsive Riboflavin‐Based Self‐Assembled Systems
Source: Chemistry. 2025 Sep 15;31(59):e00726. doi: 10.1002/chem.202500726 (PMC12548498; doi:10.1002/chem.202500726)
Supplement: Supplementary file 1 — Supporting Information [file CHEM-31-e00726-s002.docx]

**Supporting information**

**Experimental and Computational Investigation of Surface-Responsive Riboflavin-Based Self-Assembled Systems**

Ruth Aizen, Thangavel Vijayakanth, Sarah Guerin, Pierre-André Cazade, Om Shanker Tiwari, Bin Xue, Linda J. W. Shimon, Yi Cao, Damien Thompson, Ehud Gazit

| **S.N.** | **Table of Contents** | **Page No.** |
| --- | --- | --- |
| 1 | Materials and methods | 2-5 |
| 2 | Crystallographic Information | 6-10 |
| 3 | Characterization Data | 10-13 |
| 4 | SEM, AFM, MD and DFT studies | 14-17 |
| 5 | References | 18-19 |

**Materials and Methods**

Riboflavin was purchased from Sigma-Aldrich (purity > 98%) and 1,1,1,3,3,3-hexafluoro-2-propanol (HFIP) 99% was purchased from Tzamal D-Chem.

**Crystal preparation and data collection**

Crystals used for data acquisition were grown by the solvent switch method. The dry riboflavin powder was dissolved in HFIP at a concentration of 12.5 mg/ml and was then diluted in water to a concentration of 0.5mg/ml. The solution was set to rest at room temperature (23°C). Orange-colored needle-like crystals were formed within a few days. For data collection, the crystals were enveloped in paratone oil from Hampton Research, mounted on a MiTeGen cryo-loop, and swiftly frozen in liquid nitrogen. Diffraction data were subsequently collected at a temperature of 100 K using a Rigaku XtaLab^Pro^ diffractometer equipped with a Dectris PilatusR 200K-A detector, with CuKα radiation with a wavelength (λ) of 1.54184 Å.

**Processing and structural refinement of crystal data**

The diffraction data were processed utilizing CrysAlis^Pro^ 1.171.39.22a. Structure determination was achieved through direct methods using SHELXT-2016/4.^1,2^ Refinements were subsequently conducted with SHELXL-2016/4, employing weighted full-matrix least-squares against |F2| using all available data. During refinement, atoms were treated independently and anisotropically, with the exception of hydrogen atoms, which were positioned at calculated coordinates and refined in a riding mode. Details regarding crystal data collection and refinement parameters can be found in Supplementary Table 1, and the complete dataset is available in the CIF file provided as supplementary information. Crystallographic data for the structures reported in this article have been deposited with the Cambridge Crystallographic Data Centre under deposition numbers CCDC 2366098. Copies of the data can be obtained free of charge from www.ccdc.cam.ac.uk/structures.The bond distance and bond angles as well as structural illustrations were obtained using the Mercury and DIAMOND-3.1 software package.

**Atomic force microscopy (AFM) surface analysis**

Riboflavin was drop-cast on a freshly cleaved mica substrate and air-dried at room temperature. AFM images were recorded using AFM (JPK Instruments AG) with a Nano Wizard 3 using 5 N/m spring constant tips and a resonance frequency of ∼150 kHz in soft tapping mode. AFM analysis was conducted on different areas for each sample, and the height was measured using the Gwyddion 2.56 software.

**AFM nanoindentation**

Young’s modulus of the crystal was obtained using JPK, Nanowizard II (Berlin, Germany) Using a silica cantilever (PPP-SeIHR Nanosensor) with a spring constant of 10–130 N m^−1^, half-open angle of the pyramidal face of the tip was less than 10°, tip radius was ~8 nm. Sample was prepared by spreading the crystals over the surface of the quartz substrate (Supplementary Fig. S4). In a typical experiment, the cantilever was moved above the crystal with the help of optical microscope and was brought to the crystal sample at a constant speed of 2 μm s^−1^ and held to the crystal surface at a constant force of 200 nN. After retracting from the crystal surface, the cantilever was moved to the next spot to perform another cycle. The extend and retract force curves were obtained at each spot of the dot matrix using the commercial software from JPK, analyzed with an in-house software based on Igor pro 6.12 (Wavematrix 491 Inc.) and confirmed by manual fitting. 5–8 such dot matrix (5 µm × 5 µm, 600 pixels) were randomly set on each crystal. By fitting the extend and retract force curves in the range of 10 nm from the contact point using a custom-written Igor program, the Young’s modulus of each spot was calculated. The approaching curves were fitted using the Hertz model,

(1) $F\left( h \right)=\frac{2}{\pi}\tan\alpha\times\frac{E_{riboflavin/HFIP}}{1-{V^{2}}_{riboflavin/HFIP}}\times h^{2}$

where F corresponds to the stress of the cantilever, h is the depth of the indentation, α is the half angle of the tip, $E_{riboflavin/HFIP}$ is the Young’s modulus of the crystals and $v_{riboflavin/HFIP}$is the Poisson ratio which was set to 0.3. The obtained Young’s modulus was used to prepare the elasticity histogram.

**Point stiffness**

In a typical point stiffness measurement, the mechanical behavior of the crystal and cantilever was considered as linear elastic. The measured point stiffness was obtained by fitting the extend and retract force curves. Since the crystal sample and the cantilever were considered as two springs, the measured point stiffness ($K_{means}$) comprised both stiffness of the cantilever ($K_{\mathrm{can}}$) and crystal ($K_{cry}$). The point stiffness of the crystal was measured using the relation.

(2) $K_{cry}=\frac{K_{\mathrm{can}}\times K_{means}}{K_{\mathrm{can}}-K_{means}}$

**Scanning electron microscopy (SEM)**

A 7-10 μL aliquot was allowed to dry on different surfaces i.e. copper, mica, silicon wafer glass cover slip, Teflon, and Si-glass under ambient conditions overnight and coated with Au. SEM images were recorded using a ThermoFisher, Quanta 200 FEG ESEM operating at 10-20 kV.

**High resolution scanning electron microscopy (HR-SEM)**

A 7-10 μL aliquot was allowed to dry on a glass cover slip under ambient conditions overnight, and no coating was used. HRSEM images were collected using a Zeiss Gemini 300 (Zeiss, Germany) with an operating voltage of 3 kV.

**Computational methods**

A combination of density functional theory (DFT) calculations of the single crystal and classical molecular dynamics (MD) simulations of room temperature surface-bound crystalline films were used. DFT calculations were performed with VASP^3^ using the PBE exchange-correlation functional^4^ with Grimme-D3 dispersion^5^ corrections and a plane-wave cutoff of 600 eV with a 4 x4 x4 k-point sampling mesh. A finite differences method was used to calculate the stiffness tensor, with each atom being displaced in each direction by ± 0.01 Å. Piezoelectric strain constants and dielectric tensors were calculated using Density Functional Perturbation Theory^6^ (DFPT), with a plane wave cut-off of 1000 eV and k-point sampling of 2x2x2. All MD simulations were carried out using Gromacs^6^ with a time step of 2 fs for dynamics. All bonds involving a hydrogen atom were constrained with the LINCS^7^ algorithm, and the simulations were performed in the constant volume NVT ensemble. The Berendsen thermostat^8^ with a relaxation time of 1 ps was used for the heating procedure, and the V-rescale^9^ algorithm with a 1 ps relaxation time was used for equilibrium and production phases. The standard 1.2 nm cutoff was used for non-bonding interactions. The CHARMM36m^10^ forcefield was used including its dedicated small molecule counterpart CGenFF^11^ which provided parameters for riboflavin^12^. Parameters for HFIP were obtained using the ParamChem server.^13,14^ Charges were validated using the RESP procedure based on gas-phase QM calculations (B3LYP/CC-PVTZ) performed with Gaussian16^15^ software.

Systems were prepared for MD with CHARMM-GUI webserver,^16^ generating surface slabs for copper and mica. Unfortunately, there are no available parameters for pure silicon that would be compatible with CHARMM/CGenFF. Therefore, gold was used instead, as various parameter sets were derived to account for different surface properties of gold depending on the slab cut^17^. Parameters led to a surface ranging from hydrophilic, i.e., very attractive to polar adsorbates, similar to the default gold CHARMM forcefield, to hydrophobic. Both were used to describe two possible states of the silicon surface depending on the hydroxylation rate in the experiments. A 11x7x5 supercell of the crystal unit cell (measuring 59.774 Å x 248.850 Å x 59.825 Å) was suspended at a starting distance of approximately 3 Å above a four-layer slab of gold, copper, or mica. The atoms in the surface slabs were constrained around their crystallographic positions with net zero atomic charges placed on gold and copper. Copper and hydrophilicity of gold were controlled only via Lennard-Jones parameters. The mica model, however, involved covalent bonds between, Si, O, and H atoms, a set of partial charges and LJ parameters on all the atoms (Si, O, H, and Al). Therefore, unlike gold and copper, mica showed pairwise electrostatic interactions with both HFIP and riboflavin. All the slab models are part of CHARMM forcefield and freely available. The slab was periodic with unit cell surface area of ~103 Å x 281 Å, sufficiently large to host the crystal assembly. The full simulation cell was encased in a box with a large vacuum gap above the crystal with the z component fixed at 130 Å and periodic boundary conditions were applied in all directions. Each crystal:surface complex was energy minimized and then heated to 300 K and equilibrated during 1 ns of dynamics, followed by a production phase of 200 ns.

**Table S1.** Crystallographic data for riboflavin at 100 K

| **Compound** | **Riboflavin (RF)** |
| --- | --- |
| CCDC number | 2366098 |
| Chemical formula | C_20_H_22_F_6_N_4_O_7_ |
| Formula weight (g/mol) | 544.41 |
| Temperature (K) | 100 |
| Crystal system | Monoclinic |
| Space group | *P*2_1_ |
| a (Å) | 5.43380(10) |
| b (Å) | 35.5503(8) |
| c (Å) | 11.9651(3) |
| α (°) | 90 |
| β (°) | 101.177(2) |
| γ (°) | 90 |
| V (Å^3^) | 2267.50(9) |
| Z | 4 |
| Radiation | CuKα (λ = 1.54184) |
| Computing_data_collection | Rigaku XtaLab^Pro^ |
| Crystal size/mm^3^ | 0.13 × 0.05 × 0.02 |
| Absorption correction method | Multi-scan |
| ρ (calc.) g cm^-3^ | 1.595 |
| μ(Mo K_α_) mm^-1^ | 1.344 |
| F000 | 1120.0 |
| θ_max_ (°) | 80.738 |
| R(int) | 0.0799 |
| Completeness % | 99.8 |
| Data/restraints/parameters | 9583/1/689 |
| Goodness-of-fit on F^2^ | 1.059 |
| Final R indexes [I>=2σ (I)] | 0.0744, w*R*_2_ = 0.1944 |
| Final R indexes [all data] | 0.0792, w*R*_2_ = 0.1991 |
| Largest diff. peak/hole (e Å^-3^) | 0.624/-0.404 |
| Crystallization solvent | 1,1,1,3,3,3-hexafluoroisopropanol (HFIP) |

**Table S2.** Hydrogen bond distances and bond angles for riboflavin at 100 K

| **RF** | **D-H…A** | **d(D-H)** | **d(H…A)** | **d(D-A)** | **<DHA** | **Symmetry operations** |
| --- | --- | --- | --- | --- | --- | --- |
|  | N3-H3A…O7 | 0.8597 (41) | 2.0383(40) | 2.8923(57) | 172.162(275) | 3-x, -0.5+y, 3-z |
|  | N7-H7…O2 | 0.8598 (40) | 3.2090(35) | 3.0662(53) | 72.763(266) | 4-x, -0.5+y, 3-z |
|  | N7-H7…O5 | 0.8598 (40) | 2.0081(34) | 2.8335(52) | 160.601(285) | 3-x, -0.5+y, 2-z |
| **RF1** | O3-H3…O1 | 0.8198(34) | 2.0914(35) | 2.8991(50) | 168.402(265) | 4-x, -0.5+y, 2-z |
|  | O3-H3…N2 | 0.8198(34) | 2.3823(34) | 2.9777(45) | 130.173(257) | 3-x, -0.5+y, 2-z |
|  | O4-H4…O6 | 0.9590(875) | 1.7141(881) | 2.6194(57) | 169.277(7622) | 4-x, -0.5+y, 2-z |
|  | O4-H4…O13 | 0.9590(875) | 3.2085(771) | 2.6076(74) | 42.484(3865) | 3-x, -0.5+y, 2-z |
|  | O5-H5…O3 | 0.9590(875) | 1.7871(869) | 2.7279(55) | 166.137(7255) | 4-x, -0.5+y, 2-z |
|  | O6-H6…O2 | 0.7369(874) | 2.1907(770) | 2.7694(52) | 136.123(8257) | 3-x, -0.5+y, 2-z |
|  | O11-H11…O1 | 0.8199(37) | 2.5007(34) | 3.0383(51) | 124.200(293) | 3-x, -0.5+y, 3-z |
|  |  |  |  |  |  |  |
|  | N3-H3A…O7 | 0.8597 (41) | 2.0383(40) | 2.8923(57) | 172.162(275) | 3-x, -0.5+y, 3-z |
|  | N7-H7…O2 | 0.8598 (40) | 3.2090(35) | 3.0662(53) | 72.763(266) | 4-x, -0.5+y, 3-z |
|  | N7-H7…O5 | 0.8598 (40) | 2.0081(34) | 2.8335(52) | 160.601(285) | 3-x, -0.5+y, 2-z |
| **RF2** | O9-H9…N6 | 0.8201(37) | 2.2782(43) | 2.9386(58) | 137.905(271) | 3-x, -0.5+y, 3-z |
|  | O10-H10…O12 | 0.8201(33) | 1.8335(35) | 2.6477(48) | 171.731(281) | 4-x, -0.5+y, 3-z |
|  | O11-H11…O1 | 0.8199(37) | 2.5007(34) | 3.0383(51) | 124.200(293) | 4-x, -0.5+y, 2-z |
|  | O11-H11…O9 | 0.8199(37) | 2.1850(34) | 2.8469(52) | 137.893(309) | 5-x, -0.5+y, 3-z |
|  | O12-H12…O8 | 0.9533(1043) | 1.7999(1033) | 2.7468(58) | 171.80(921) | 4-x, -0.5+y, 4-z |
|  | O14-H14…O10 | 0.8199(84) | 3.0740(36) | 2.5770(68) | 46.321(483) | 4-x, -0.5+y, 3-z |

**Table S3.** Bond lengths [Å] for riboflavin

| **Atom 1** | **Atom 2** | **Bond length** | **Atom 1** | **Atom 2** | **Bond length** |
| --- | --- | --- | --- | --- | --- |
| O1 | C2 | 1.233(6) | O9 | H9A | 0.82 |
| O2 | C3 | 1.227(6) | O9 | C31 | 1.409(6) |
| O3 | H3 | 0.82 | O10 | H10 | 0.82 |
| O3 | C14 | 1.418(6) | O10 | C32 | 1.424(6) |
| O4 | H4 | 0.92(9) | O11 | H11 | 0.82 |
| O4 | C15 | 1.437(6) | O11 | C33 | 1.428(7) |
| O5 | H5 | 0.96(9) | O12 | H12 | 1.0(1) |
| O5 | C16 | 1.432(6) | O12 | C34 | 1.431(7) |
| O6 | H6 | 0.74(9) | N5 | C18 | 1.369(6) |
| O6 | C17 | 1.414(6) | N5 | C27 | 1.397(7) |
| N1 | C1 | 1.366(7) | N5 | C28 | 1.477(6) |
| N1 | C10 | 1.386(6) | N6 | C18 | 1.321(7) |
| N1 | C13 | 1.470(6) | N6 | C19 | 1.362(6) |
| N2 | C1 | 1.310(6) | N7 | H7 | 0.86 |
| N2 | C2 | 1.366(7) | N7 | C19 | 1.387(6) |
| N3 | H3A | 0.86 | N7 | C20 | 1.366(7) |
| N3 | C2 | 1.404(6) | N8 | C21 | 1.309(7) |
| N3 | C3 | 1.359(6) | N8 | C22 | 1.365(6) |
| N4 | C4 | 1.295(6) | C18 | C21 | 1.441(7) |
| N4 | C5 | 1.379(7) | C20 | C21 | 1.486(7) |
| C1 | C4 | 1.438(6) | C22 | C23 | 1.419(8) |
| C3 | C4 | 1.490(7) | C22 | C27 | 1.406(7) |
| C5 | C6 | 1.397(7) | C23 | C24 | 1.374(7) |
| C5 | C10 | 1.411(6) | C24 | C25 | 1.431(9) |
| C6 | C7 | 1.387(8) | C24 | C029 | 1.506(9) |
| C7 | C8 | 1.425(8) | C25 | C26 | 1.399(8) |
| C7 | C11 | 1.494(7) | C25 | C30A | 1.484(8) |
| C8 | C9 | 1.382(7) | C26 | C27 | 1.400(7) |
| C8 | C12 | 1.503(8) | C28 | C31 | 1.530(7) |
| C9 | C10 | 1.409(7) | O14 | H14A | 0.82 |
| O7 | C19 | 1.236(7) | O13 | H13 | 0.82 |
| O8 | C20 | 1.219(6) |  |  |  |

**Table S4.** Bond angles [°] for riboflavin

| **Atom 1** | **Atom 2** | **Atom 3** | **Bond angle** | **Atom 1** | **Atom 2** | **Atom 3** | **Bond angle** |
| --- | --- | --- | --- | --- | --- | --- | --- |
| C1 | N1 | C10 | 120.6(4) | C27 | N5 | C28 | 121.7(4) |
| C1 | N1 | C13 | 117.0(4) | C18 | N6 | C19 | 118.5(4) |
| C10 | N1 | C13 | 122.3(4) | C19 | N7 | C20 | 125.6(4) |
| C1 | N2 | C2 | 118.8(4) | C21 | N8 | C22 | 116.9(4) |
| C2 | N3 | C3 | 125.5(4) | N5 | C18 | N6 | 118.6(4) |
| C4 | N4 | C5 | 117.0(4) | N5 | C18 | C21 | 117.2(4) |
| N1 | C1 | N2 | 118.7(4) | N6 | C18 | C21 | 124.2(4) |
| N1 | C1 | C4 | 116.4(4) | O7 | C19 | N6 | 120.9(4) |
| N2 | C1 | C4 | 124.8(4) | O7 | C19 | N7 | 118.6(4) |
| O1 | C2 | N2 | 121.5(4) | N6 | C19 | N7 | 120.5(4) |
| O1 | C2 | N3 | 118.8(4) | O8 | C20 | N7 | 122.5(5) |
| N2 | C2 | N3 | 119.7(4) | O8 | C20 | C21 | 123.8(5) |
| O2 | C3 | N3 | 122.8(5) | N7 | C20 | C21 | 113.7(4) |
| O2 | C3 | C4 | 123.1(4) | N8 | C21 | C18 | 124.6(5) |
| N3 | C3 | C4 | 114.1(4) | N8 | C21 | C20 | 118.1(4) |
| N4 | C4 | C1 | 125.5(4) | C18 | C21 | C20 | 117.3(4) |
| N4 | C4 | C3 | 117.5(4) | N8 | C22 | C23 | 117.4(4) |
| C1 | C4 | C3 | 116.9(4) | N8 | C22 | C27 | 123.1(4) |
| N4 | C5 | C6 | 118.4(4) | C23 | C22 | C27 | 119.5(5) |
| N4 | C5 | C10 | 121.8(4) | C22 | C23 | C24 | 121.6(5) |
| C6 | C5 | C10 | 119.8(4) | C23 | C24 | C25 | 118.8(5) |
| C5 | C6 | C7 | 122.2(5) | C23 | C24 | C029 | 120.6(5) |
| C6 | C7 | C8 | 117.8(5) | C25 | C24 | C029 | 120.6(5) |
| C6 | C7 | C11 | 120.8(5) | C24 | C25 | C26 | 119.9(5) |
| C8 | C7 | C11 | 121.4(5) | C24 | C25 | C30A | 121.0(5) |
| C7 | C8 | C9 | 120.6(5) | C26 | C25 | C30A | 119.1(5) |
| C7 | C8 | C12 | 120.1(5) | C25 | C26 | C27 | 120.8(5) |
| C9 | C8 | C12 | 119.3(5) | N5 | C27 | C22 | 118.2(4) |
| C8 | C9 | C10 | 121.2(5) | N5 | C27 | C26 | 122.5(4) |
| N1 | C10 | C5 | 118.6(4) | C22 | C27 | C26 | 119.3(5) |
| N1 | C10 | C9 | 122.9(4) | N5 | C28 | C31 | 110.8(4) |
| C5 | C10 | C9 | 118.4(4) | O9 | C31 | C28 | 111.2(4) |
| N1 | C13 | C14 | 112.5(4) | O9 | C31 | C32 | 106.6(4) |
| C18 | N5 | C27 | 120.0(4) | C28 | C31 | C32 | 112.9(4) |
| C18 | N5 | C28 | 118.4(4) |  |  |  |  |

**Table S5.** Torsion angles [°] for riboflavin

| **Atom 1** | **Atom 2** | **Atom 3** | **Atom 4** | **Torsion angle** | **Atom 1** | **Atom 2** | **Atom 3** | **Atom 4** | **Torsion angle** |
| --- | --- | --- | --- | --- | --- | --- | --- | --- | --- |
| C1 | N1 | C13 | H13A | 152.9 | N1 | C13 | C14 | H14 | 71.5 |
| C1 | N1 | C13 | C14 | -85.9(5) | N1 | C13 | C14 | C15 | -167.9(4) |
| C1 | N2 | C2 | O1 | 173.3(4) | C27 | N5 | C18 | N6 | 178.6(4) |
| C2 | N2 | C1 | N1 | -174.8(4) | C28 | N5 | C18 | C21 | -179.8(4) |
| C2 | N3 | C3 | O2 | 175.9(5) | C18 | N5 | C27 | C26 | -179.6(5) |
| C3 | N3 | C2 | O1 | -173.3(4) | C28 | N5 | C27 | C22 | -179.8(4) |
| C4 | N4 | C5 | C6 | 177.4(4) | C18 | N5 | C28 | H28A | 153.4 |
| C5 | N4 | C4 | C3 | -176.1(4) | C18 | N5 | C28 | H28B | 35.1 |
| C6 | C7 | C11 | H11A | -107.5 | C18 | N5 | C28 | C31 | -85.8(5) |
| C6 | C7 | C11 | H11B | 132.5 | C27 | N5 | C28 | H28B | -144.3 |
| C7 | C8 | C9 | H9 | -179.1 | C27 | N5 | C28 | C31 | 94.8(5) |
| C8 | C7 | C11 | H11C | -168.3 | N5 | C18 | C21 | C20 | 177.9(4) |
| C9 | C8 | C12 | H12A | 108.5 | N6 | C18 | C21 | N8 | -179.8(5) |
| C9 | C8 | C12 | H12C | -131.6 | O8 | C20 | C21 | C18 | -178.2(5) |
| N1 | C1 | C4 | C3 | 177.1(4) | N7 | C20 | C21 | N8 | 179.8(4) |
| N2 | C1 | C4 | N4 | -177.7(5) | N8 | C22 | C23 | C24 | -177.0(5) |
| N3 | C3 | C4 | N4 | 177.6(4) | C27 | C22 | C23 | H23 | -178.2 |
| N4 | C5 | C6 | C7 | -177.4(5) | N8 | C22 | C27 | C26 | -179.8(5) |
| N4 | C5 | C10 | C9 | 179.1(4) | O14 | C39 | C40 | F11 | 56.8(9) |
| C12 | C8 | C9 | C10 | -177.6(5) | O14 | C39 | C40 | F10 | -62.5(9) |
| N1 | C13 | C14 | O3 | -49.9(5) | O14 | C39 | C40 | F12 | 179.4(7) |


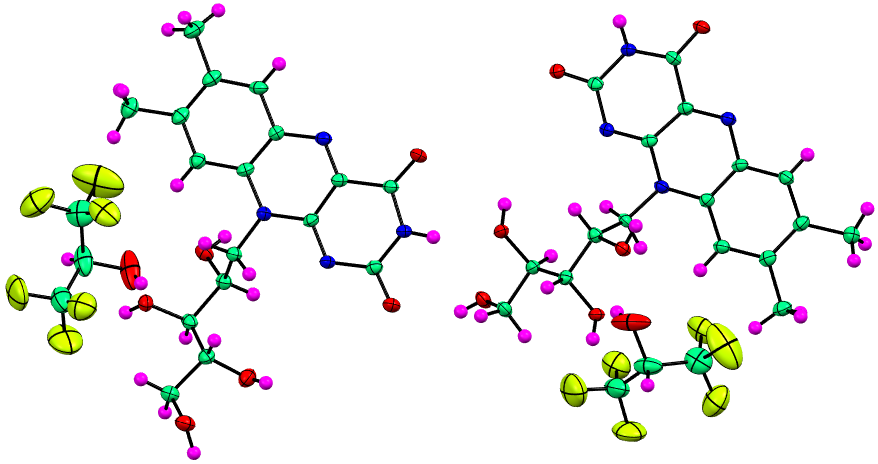


**Figure S1.** ORTEP diagram of the asymmetric unit of riboflavin with the thermal ellipsoids drawn at 50% probability. The carbon, hydrogen, nitrogen, oxygen and fluorine atoms are represented as spring green, magenta, blue, red and yellow-green, respectively.


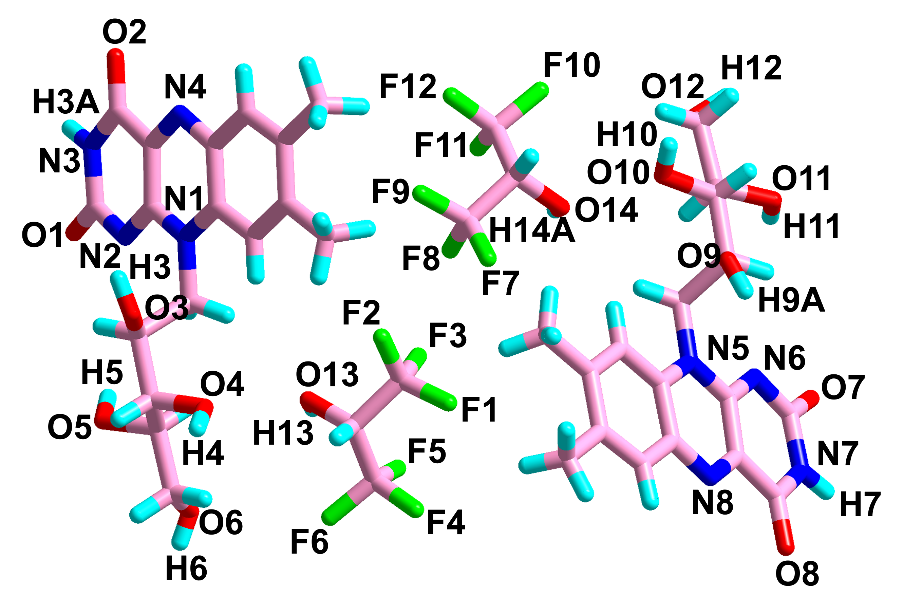


**Figure S2.** Asymmetric unit including atom numbering.


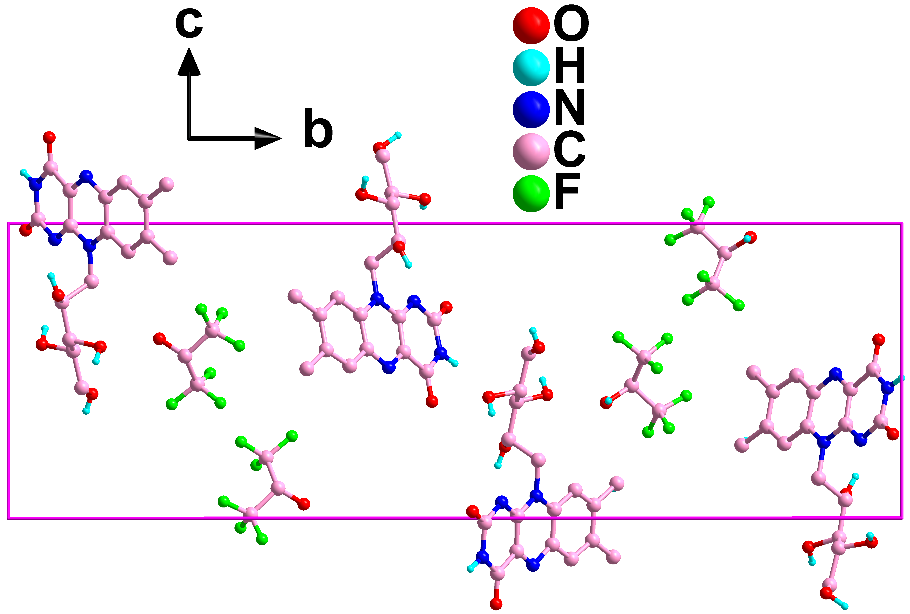


**Figure S3.** Representation of crystal packing unit-cell diagram of riboflavin at 100K. The carbon, hydrogen, nitrogen, oxygen, and fluorine atoms are represented as rose, turquoise, blue, red and green, respectively.

**Figure S4.** Higher-order hydrogen-bonded assemblies involving the isoalloxazine ring of each riboflavin molecule and HFIP molecule.

**Figure S5.** A projection of (a) intra- and (b) intermolecular H-bonded structures of RF1 and RF2. The dashed lines are depicted to display the H-bonded interactions. The carbon, hydrogen, nitrogen and oxygen atoms are represented as rose, turquoise, blue, red and green, respectively.


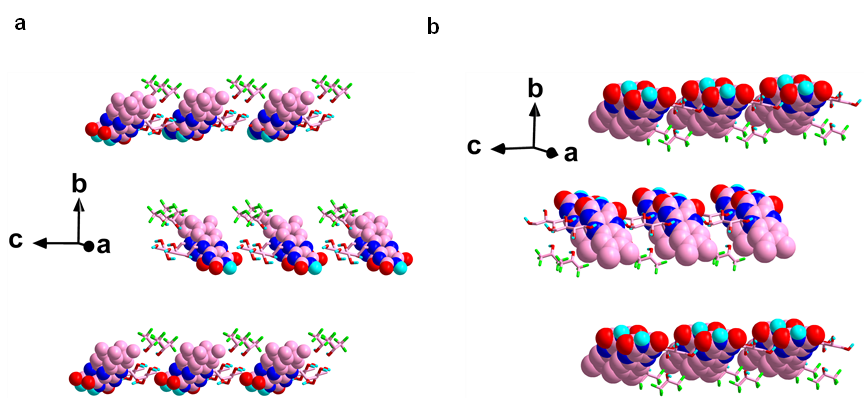


**Figure S6.** (a and b) Representation of the aromatic-aromatic interactions between two isoalloxazine rings of the RF1 and RF2. The highlighted space-filling model illustrates the molecular arrangement of the isoalloxazine rings. The carbon, hydrogen, nitrogen, oxygen, and fluorine atoms are represented as rose, turquoise, blue, red and green, respectively.

**
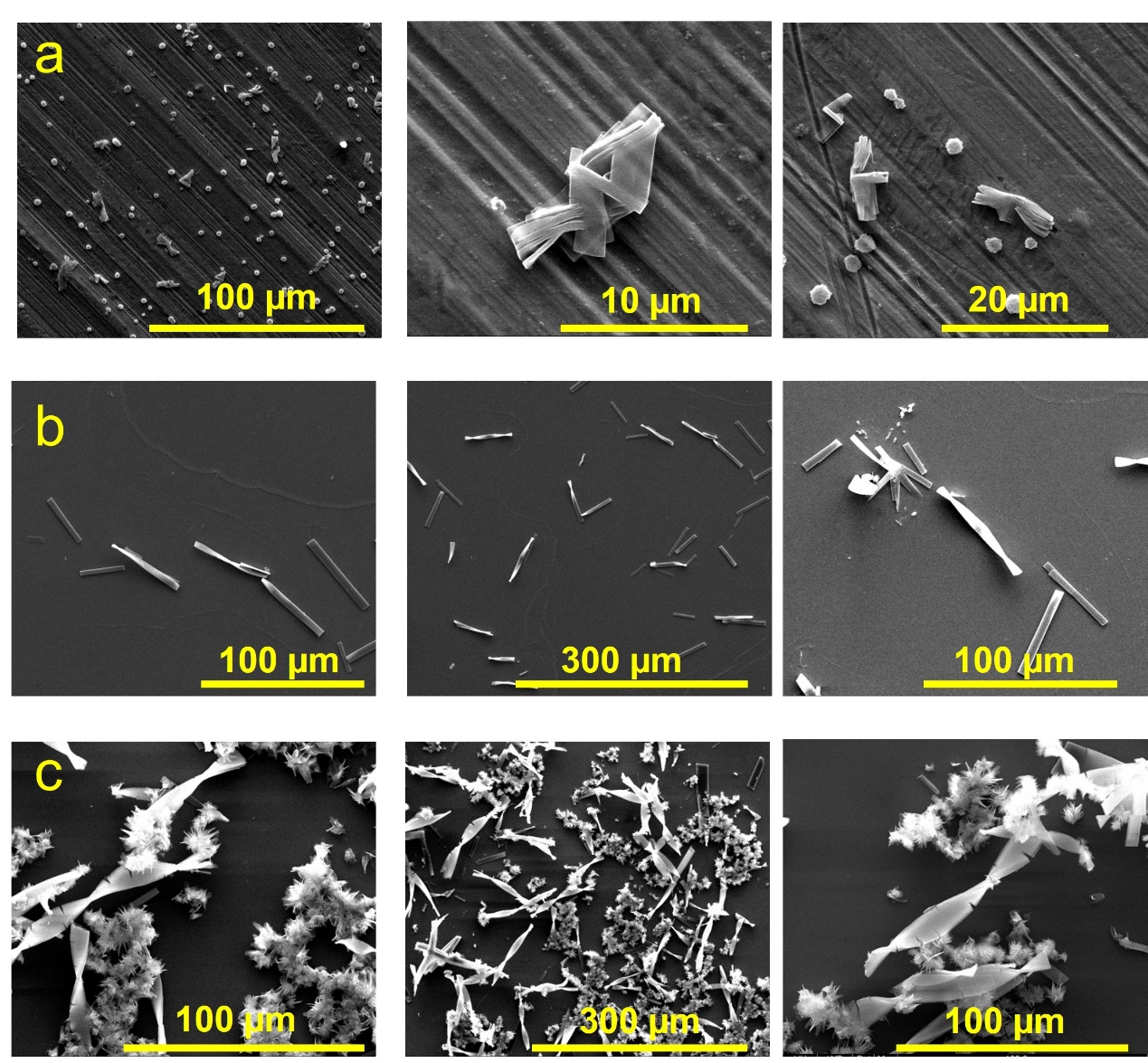
**

**Figure S7**. Additional SEM images of riboflavin/HFIP crystals deposited on three different surfaces. (a) copper, (b) mica, (c) silicon.

**Figure S8**. SEM images of riboflavin/HFIP crystals deposited on three different surfaces. (a) Teflon. (b) Si-glass. (c) Glass.

**Figure S9**. Electrostatic and van der Waals interaction energy per molecule for the four surfaces tested: mica (black), copper (red), hydrophobic gold (AuO, green), and hydrophilic gold (AuY, blue). AuO provides a model for bare hydrophobic Si and AuY provides an additional model for a pure-metal hydrophilic substrate. (a) Interaction energy between the surface and riboflavin. (b) Interaction energy between the surface and HFIP. (c) Interaction energy between riboflavin and HFIP. (d) Self-interaction of riboflavin. (e) Self-interaction of HFIP.

**
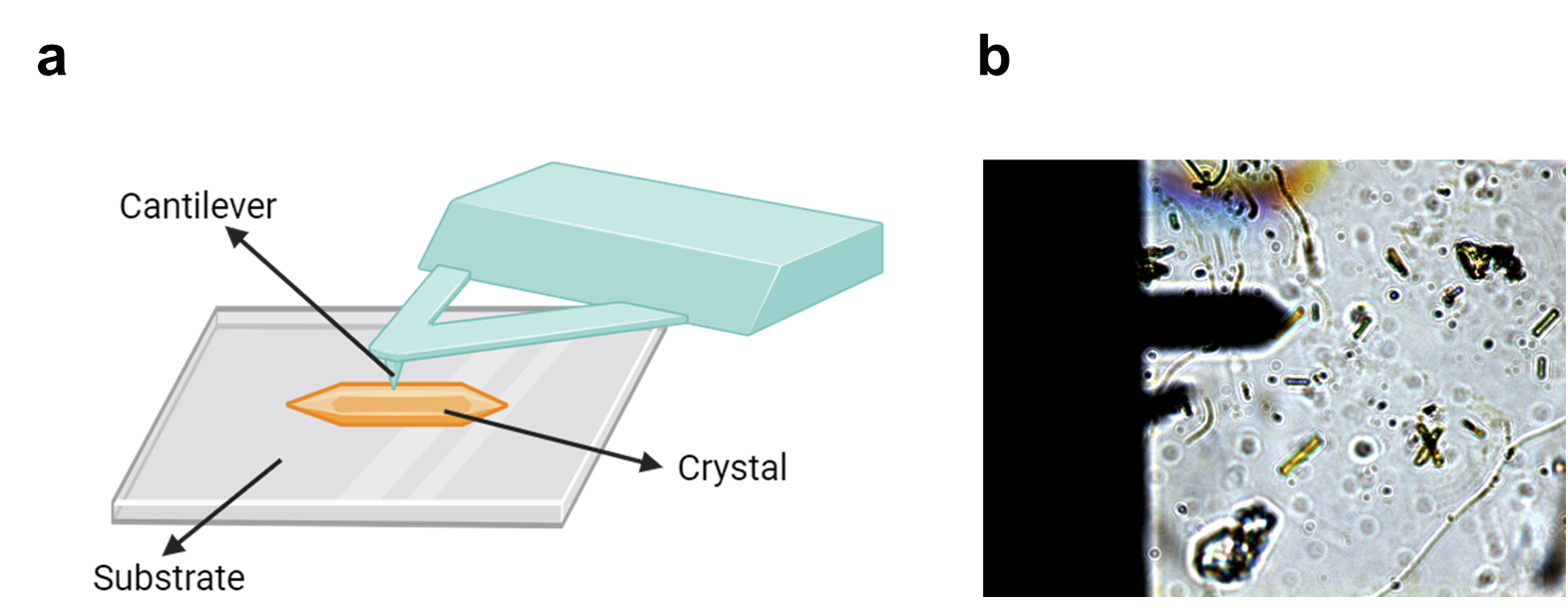
**

**Figure S10**. (a) Schematic illustration of the AFM experiments. (b) A typical IT-AFM image. The crystals were fixed to the surface of a glass coverslip and the cantilever was moved to the crystal surface while monitoring under an optical microscope.

**Table S6.** Elastic constants of riboflavin/HFIP crystals predicted using DFT calculations.

| **Elastic Stiffness Constant** | **(GPa)** |
| --- | --- |
| c_11_ | 13.6 |
| c_22_ | 17.4 |
| c_33_ | 32.5 |
| c_44_ | 2.5 |
| c_55_ | 7.3 |
| c_66_ | 2.2 |
| Young’s Modulus | 7.6 ± 3.8 |


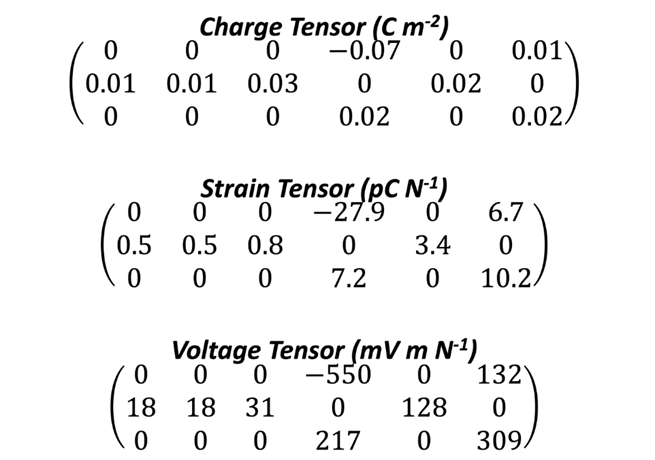


**Figure S11.** Piezoelectric responses of the riboflavin/HFIP crystal predicted using DFT calculations. Results are shown as charge, strain, and voltage tensors.

**Table S7.** Dielectric constants of riboflavin/HFIP crystals predicted using DFT calculations.

| **Dielectric Constant** | **(unitless)** |
| --- | --- |
| ε_1_ | 5.7 |
| ε_2_ | 3.0 |
| ε_3_ | 3.7 |
| ε_r_ | 4.1 |

**References:**

1. G. M. Sheldrick, *Acta Crystallogr. Sect. A* **2008,** *64,* 112–122.
2. A. L. Spek, *Acta Crystallogr. Sect. D* **2009,** *65,* 148–155.
3. G. Kresse, J. Furthmüller, *Phys. Rev. B* **1996,** *54*, 11169–11186.
4. J. P. Perdew, K. Burke, M. Ernzerhof, *Phys. Rev. Lett.* **1996,** *77*, 3865–3868.
5. S. Grimme, J. Antony, S. Ehrlich, H. A. Krieg, *J. Chem. Phys.* **2010,** *132,* 154104.
6. D. Van der Spoel, E. Lindahl, B. Hess, G. Groenhof, A. E. Mark, H. J. C. Berendsen, *J. Comput. Chem.* **2005,** *26,* 1701–1718.
7. B. Hess, *J. Chem. Theory Comput.* **2008,** *4,* 116–122.
8. H. J. C. Berendsen, J. P. M. Postma, W. F. van Gunsteren, A. DiNola, J. R. Haak, *J. Chem. Phys.* **1984,** *81,* 3684–3690.
9. G. Bussi, D. Donadio, M. Parrinello, *J. Chem. Phys.* **2007,** *126,* 014101.
10. J. Huang, S. Rauscher, G. Nawrocki, T. Ran, M. Feig, B. L. de Groot, H. Grubmüller, A. D. MacKerell Jr, *Nat. Methods* **2017,** *14,* 71–73.
11. K. Vanommeslaeghe, E. Hatcher, C. Acharya, S. Kundu, S. Zhong, J. Shim, E. Darian, O. Guvench, P. Lopes, I. Vorobyov, A. D. Mackerell Jr, *J. Comput. Chem.* **2010,** *31,* 671–690.
12. A. Aleksandrov, *J. Comput. Chem.* **2019,** *40,* 2834–2842.
13. K. Vanommeslaeghe, A. D. MacKerell, *J. Chem. Inf. Model.* **2012,** *52,* 3144–3154.
14. K. Vanommeslaeghe, E. P. Raman, A. D. MacKerell, *J. Chem. Inf. Model.* **2012,** *52,* 3155–3168.
15. M. J. Frisch, G. W. Trucks, H. B. Schlegel, G. E. Scuseria, M. A. Robb, J. R. Cheeseman, G. Scalmani, V. Barone, G. A. Petersson, H. Nakatsuji, X. Li, M. Caricato, A. V. Marenich, J. Bloino, B. G. Janesko, R. Gomperts, B. Mennucci, H. P. Hratchian, J. V. Ortiz, A. F. Izmaylov, J. L. Sonnenberg, D. Williams-Young, F. Ding, F. Lipparini, F. Egidi, J. Goings, B. Peng, A. Petrone, T. Henderson, D. Ranasinghe, V. G. Zakrzewski, J. Gao, N. Rega, G. Zheng, W. Liang, M. Hada, M. Ehara, K. Toyota, R. Fukuda, J. Hasegawa, M. Ishida, T. Nakajima, Y. Honda, O. Kitao, H. Nakai, T. Vreven, K. Throssell, J. A. Montgomery, Jr., J. E. Peralta, F. Ogliaro, M. J. Bearpark, J. J. Heyd, E. N. Brothers, K. N. Kudin, V. N. Staroverov, T. A. Keith, R. Kobayashi, J. Normand, K. Raghavachari, A. P. Rendell, J. C. Burant, S. S. Iyengar, J. Tomasi, M. Cossi, J. M. Millam, M. Klene, C. Adamo, R. Cammi, J. W. Ochterski, R. L. Martin, K. Morokuma, O. Farkas, J. B. Foresman, and D. J. Fox, Gaussian, Inc., Wallingford CT, **2016.**
16. J. Lee, X. Cheng, J. M. Swails, M. S. Yeom, P. K. Eastman, J. A. Lemkul, S. Wei, J. Buckner, J. C. Jeong, Y. Qi, S. Jo, V. S. Pande, D. A. Case, C. L. Brooks III, A. D. MacKerell Jr, J. B. Klauda, W. Im, *J. Chem. Theory Comput.* **2016,** *12,* 405–413.
17. G. Nawrocki, M. Cieplak, *J. Phys. Chem. C* **2014,** *118,* 12929–12943.
