# Supplementary material for: Experimental and Computational Investigation of Surface‐Responsive Riboflavin‐Based Self‐Assembled Systems
Source: Chemistry. 2025 Sep 15;31(59):e00726. doi: 10.1002/chem.202500726 (PMC12548498; doi:10.1002/chem.202500726)

checkCIF/PLATON report

You have not supplied any structure factors. As a result the full set of tests cannot be run.

THIS REPORT IS FOR GUIDANCE ONLY. IF USED AS PART OF A REVIEW PROCEDURE FOR PUBLICATION, IT SHOULD NOT REPLACE THE EXPERTISE OF AN EXPERIENCED CRYSTALLOGRAPHIC REFEREE.

No syntax errors found.   CIF dictionary   Interpreting this report

Datablock: riboflavin_HFIP

```
Bond precision:  C-C = 0.0080 A          Wavelength=1.54184

Cell:            a=5.4338(1)    b=35.5503(8)       c=11.9651(3)
                 alpha=90       beta=101.177(2)    gamma=90
Temperature:     100 K

                Calculated                 Reported
Volume          2267.50(9)                 2267.50(9)
Space group     P 21                       P 21
Hall group      P 2yb                      P 2yb
Moiety formula  C17 H20 N4 O6, C3 H2 F6 O  C20 H22 F6 N4 O7
Sum formula     C20 H22 F6 N4 O7           C20 H22 F6 N4 O7
Mr              544.42                     544.41
Dx,g cm-3       1.595                      1.595
Z               4                          4
Mu (mm-1)       1.344                      1.344
F000            1120.0                     1120.0
F000'           1124.88
h,k,lmax        6,45,15                    6,44,15
Nref            9917[ 5040]                9583
Tmin,Tmax       0.928,0.971                0.861,1.000
Tmin'           0.842

Correction method= # Reported T Limits: Tmin=0.861 Tmax=1.000
AbsCorr = MULTI-SCAN

Data completeness= 1.90/0.97      Theta(max)= 80.738

R(reflections)= 0.0744( 8631)     wR2(reflections)= 0.1991( 9583)

S = 1.059                  Npar= 689
```

The following ALERTS were generated. Each ALERT has the format
test-name_ALERT_alert-type_alert-level.
Click on the hyperlinks for more details of the test.

Alert level C

```
PLAT089_ALERT_3_C Poor Data / Parameter Ratio (Zmax < 18) ........       7.31 Note
PLAT250_ALERT_2_C Large U3/U1 Ratio for Average U(i,j) Tensor ....        2.3 Note
PLAT340_ALERT_3_C Low Bond Precision on  C-C Bonds ...............    0.00797 Ang.
PLAT790_ALERT_4_C Centre of Gravity not Within Unit Cell: Resd.  #          1 Note
              C17 H20 N4 O6
```

Alert level G

```
PLAT007_ALERT_5_G Number of Unrefined Donor-H Atoms ..............          8 Report
PLAT042_ALERT_1_G Calc. and Reported MoietyFormula Strings  Differ     Please Check
PLAT072_ALERT_2_G SHELXL First  Parameter in WGHT  Unusually Large       0.14 Report
PLAT242_ALERT_2_G Low    'MainMol' Ueq as Compared to Neighbors of        C35 Check
PLAT242_ALERT_2_G Low    'MainMol' Ueq as Compared to Neighbors of        C37 Check
PLAT242_ALERT_2_G Low    'MainMol' Ueq as Compared to Neighbors of        C38 Check
PLAT242_ALERT_2_G Low    'MainMol' Ueq as Compared to Neighbors of        C40 Check
PLAT432_ALERT_2_G Short Inter X...Y Contact  O2       ..C19              2.91 Ang.
PLAT432_ALERT_2_G Short Inter X...Y Contact  C2       ..C5               3.18 Ang.
PLAT720_ALERT_4_G Number of Unusual/Non-Standard Labels ..........          1 Note
PLAT790_ALERT_4_G Centre of Gravity not Within Unit Cell: Resd.  #          2 Note
              C17 H20 N4 O6
PLAT790_ALERT_4_G Centre of Gravity not Within Unit Cell: Resd.  #          4 Note
              C3 H2 F6 O
PLAT791_ALERT_4_G Model has Chirality at C14         (Chiral SPGR)          S Verify
PLAT791_ALERT_4_G Model has Chirality at C15         (Chiral SPGR)          S Verify
PLAT791_ALERT_4_G Model has Chirality at C16         (Chiral SPGR)          R Verify
PLAT791_ALERT_4_G Model has Chirality at C31         (Chiral SPGR)          S Verify
PLAT791_ALERT_4_G Model has Chirality at C32         (Chiral SPGR)          S Verify
PLAT791_ALERT_4_G Model has Chirality at C33         (Chiral SPGR)          R Verify
PLAT933_ALERT_2_G Number of OMIT Records in Embedded .res File ...          7 Note
```

---

 0 **ALERT level A** = Most likely a serious problem - resolve or explain
 0 **ALERT level B** = A potentially serious problem, consider carefully
 4 **ALERT level C** = Check. Ensure it is not caused by an omission or oversight
19 **ALERT level G** = General information/check it is not something unexpected

 1 ALERT type 1 CIF construction/syntax error, inconsistent or missing data
 9 ALERT type 2 Indicator that the structure model may be wrong or deficient
 2 ALERT type 3 Indicator that the structure quality may be low
10 ALERT type 4 Improvement, methodology, query or suggestion
 1 ALERT type 5 Informative message, check

It is advisable to attempt to resolve as many as possible of the alerts in all categories. Often the minor alerts point to easily fixed oversights, errors and omissions in your CIF or refinement strategy, so attention to these fine details can be worthwhile. In order to resolve some of the more serious problems it may be necessary to carry out additional measurements or structure refinements. However, the purpose of your study may justify the reported deviations and the more serious of these should normally be commented upon in the discussion or experimental section of a paper or in the "special_details" fields of the CIF. checkCIF was carefully designed to identify outliers and unusual parameters, but every test has its limitations and alerts that are not important in a particular case may appear. Conversely, the absence of alerts does not guarantee there are no aspects of the results needing attention. It is up to the individual to critically assess their own results and, if necessary, seek expert advice.

Publication of your CIF in IUCr journals

A basic structural check has been run on your CIF. These basic checks will be run on all CIFs submitted for publication in IUCr journals (*Acta Crystallographica*, *Journal of Applied Crystallography*, *Journal of Synchrotron Radiation*); however, if you intend to submit to *Acta Crystallographica Section C* or *E* or *IUCrData*, you should make sure that full publication checks are run on the final version of your CIF prior to submission.

Publication of your CIF in other journals

Please refer to the *Notes for Authors* of the relevant journal for any special instructions relating to CIF submission.

PLATON version of 23/04/2018; check.def file version of 23/04/2018

Datablock riboflavin_HFIP - ellipsoid plot

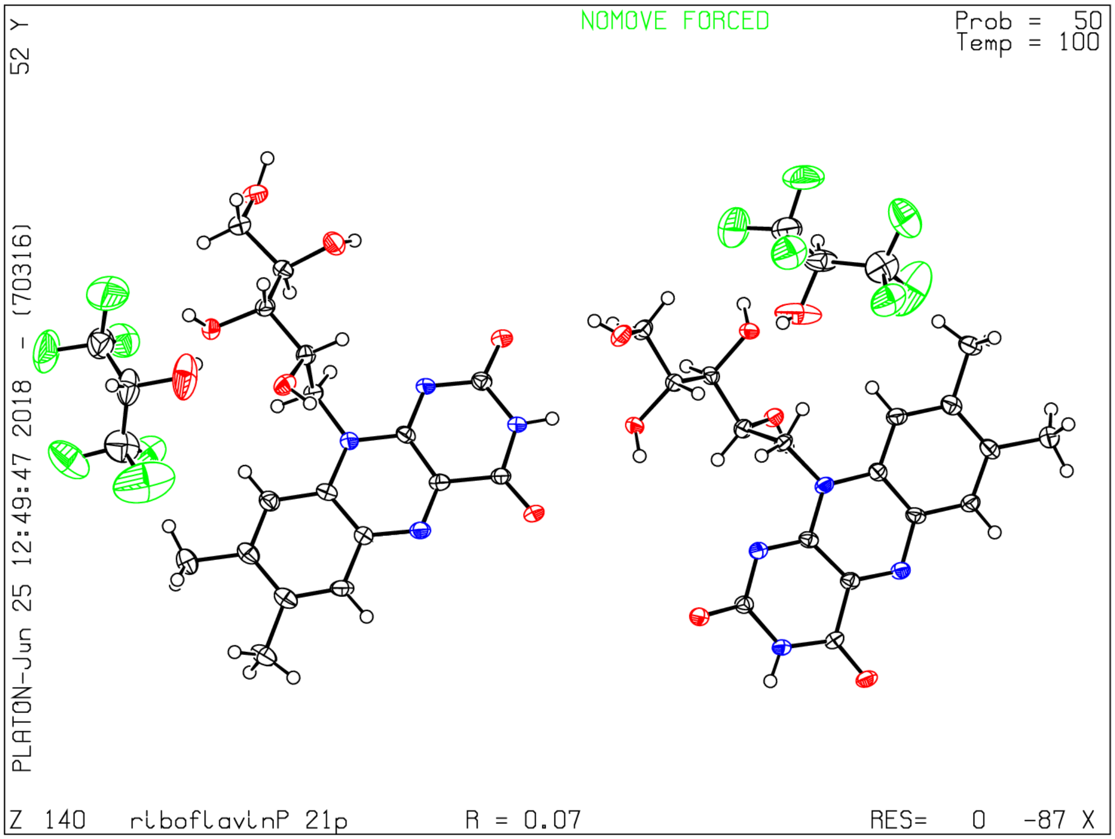

Supplement: Supplementary file 2 — Supporting Information [file CHEM-31-e00726-s001.zip › eh100riboflavincheckcif.pdf]
